# Multi-Walled Carbon Nanotubes (MWCNTs) Cause Cellular Senescence in TGF-β Stimulated Lung Epithelial Cells

**DOI:** 10.3390/toxics9060144

**Published:** 2021-06-19

**Authors:** Joseph H. Lucas, Qixin Wang, Thivanka Muthumalage, Irfan Rahman

**Affiliations:** Department of Environmental Medicine, School of Medicine and Dentistry, University of Rochester Medical Center, Rochester, NY 14642, USA; Joseph_Lucas@URMC.Rochester.edu (J.H.L.); Qixin_Wang@URMC.Rochester.edu (Q.W.); Thivanka_Muthumalage@URMC.Rochester.edu (T.M.)

**Keywords:** MWCNT, senescence, pulmonary fibrosis, epithelial cell

## Abstract

Multi-walled carbon nanotubes are engineered nanomaterials (ENMs) that have a fiber-like structure which may be a concern for the development of cellular senescence. Premature senescence, a state of irreversible cell cycle arrest, is implicated in the pathogenesis of chronic lung diseases such as pulmonary fibrosis (PF). However, the crosstalk between downstream pathways mediating fibrotic and senescent responses of MWCNTs is not well-defined. Here, we exposed human bronchial epithelial cells (BEAS-2B) to MWCNTs for up to 72 h and demonstrate that MWCNTs increase reactive oxygen species (ROS) production accompanied by inhibition of cell proliferation. In addition, MWCNT exposure resulted in the increase of p21 protein abundance and senescence associated β-galactosidase (SA β-gal) activity. We also determined that co-exposure with the cytokine, transforming growth factor-β (TGF-β) exacerbated cellular senescence indicated by increased protein levels of p21, p16, and γH2A.X. Furthermore, the production of fibronectin and plasminogen activator inhibitor (PAI-1) was significantly elevated with the co-exposure compared to MWCNT or TGF-β alone. Together, our study suggests that the cellular senescence potential of MWCNTs may be enhanced by pro-fibrotic mediators, such as TGF-β in the surrounding microenvironment.

## 1. Introduction

Multi-walled carbon nanotubes (MWCNTs) are fiber-like engineered nanomaterials (ENMs) with unique physiochemical properties that have promising usage in a variety of biomedical applications including tissue scaffolding, drug delivery systems, and biosensors [1]. Production of MWCNTs has risen rapidly over the past decade, prompting public health concerns regarding their possible adverse health effects [2]. One of the main concerns with exposure to fiber-like ENMs is pulmonary fibrosis (PF). PF is a progressive interstitial lung disease that arises from excessive deposition and production of extracellular matrix (ECM) proteins by activated myofibroblasts. Due to their small diameter (<100 nm), MWCNTs can penetrate into the deeper regions of the lung. In addition, their chemical stability and high aspect ratio impedes phagocytosis and clearance by lung macrophages, resulting in biopersistence [3]. In rodent studies, inhalation of MWCNTs triggers airway inflammation and can potentiate fibrotic responses [4], implicating MWCNTs in the development of PF and other chronic lung diseases. However, the mechanisms underlying the MWCNT induced fibrotic response is not fully understood.

Cellular senescence, a state of irreversible growth arrest, is implicated in both aging and the development of age-associated chronic lung diseases such as PF. Analysis of lung tissues from patients shows increased levels of established senescence biomarkers such as p21, p16, and senescence associated β-galactosidase (SA β-gal) [5,6]. Premature senescence can be induced by a variety of pro-aging stressors including redox imbalance, telomere shortening, and genomic instability [6]. Moreover, the adoption of a secretome known as the senescence associated secretory phenotype (SASP) results in the release of a plethora of cytokines, growth factors, and matrix metalloproteinases that drive pathologic lung remodeling and inflammation [7]. The involvement of senescence in PF is further supported by the use of senolytic drugs [8]. The clearance of senescence cells has been shown to improve pulmonary function and inhibit the fibrotic secretome in rodents in vivo [6,9]. A clinical trial also demonstrated that administration of senolytics to PF patients moderately improved patient clinical scores in mobility assessments [10]. Together, these studies demonstrate a clear role for senescence in the development of PF.

Many reports indicate that exposure to MWCNTs can lead to the development of pulmonary fibrosis in vivo [4,11,12] and drive pro-fibrotic epithelial-mesenchymal transition (EMT), and myofibroblast differentiation and activation in vitro [13,14]. Previous studies also indicate that certain MWCNTs can result in increased oxidative stress and inflammation which play vital roles in PF. Pro-inflammatory cytokines such as interleukin-8 (IL-8) are secreted from both the epithelium and resident immune cells in response to MWCNT exposure [15,16]. IL-8 is a potent neutrophil chemoattractant that is elevated in PF [17]. Recruited neutrophils promote the activation of fibroblasts and contribute to the turnover of the extracellular matrix in both normal and pathologic wound healing [18]. Furthermore, IL-8 is consistently observed in SASP [19] and paracrine signaling by neutrophils has been demonstrated to cause cellular senescence [20,21]. However, the specific role of cellular senescence in MWCNT induced fibrotic responses is poorly understood.

MWCNTs are often studied as a single exposure within a homeostatic system. While these studies can be helpful for identifying whether environmental exposure results in disease, they may mask the effects of less potent environmental exposures. Moreover, they fail to account for exposures that affect susceptible populations. Understanding the potential adverse effects of MWCNT exposure alone and in association with other fibrotic factors is important to establish risk. Thus, we utilized TGF-β as a second fibrotic stimuli to investigate the ability of MWCNTs to modulate an established fibrotic response. We hypothesized that MWCNTs would cause cellular senescence that would be exacerbated under pro-fibrotic conditions. Here, we show that MWCNT exposure modestly increases senescence biomarkers under normal conditions. We further show that exposure to MWCNTs and TGF-β more strongly induced cellular senescence. Together, our results suggest that crosstalk between pro-fibrotic and pro-senescence pathways mediates the adverse effects of MWCNT exposure.

## 2. Materials and Methods

### 2.1. MWCNT Preparation and Characterization

MWCNTs (Cat#: 030101, Cheap Tubes, Grafton, VA, USA) were suspended in dispersion media formulated by Porter et al. [22] (0.6 mg/mL BSA, 5.5 mM D-glucose, and 0.01 mg/mL dipalmitoylphosphatidylcholine in PBS) and probe sonicated for 10 min to create a stock solution (2 mg/mL). MWCNTs were probe sonicated before each treatment for 5 min and diluted in serum free culture media to desired concentrations for exposures. Dispersion media was diluted in culture media for vehicle controls and had no effect on any measured parameters. MWCNT physical characteristics are provided by the manufacturer. MWCNTs diluted in cell culture media were assessed for mean hydrodynamic radius (Z-average), polydispersity index (PdI), and zeta potential using a Malvern Zetasizer Nano ZS instrument (Malvern Panalytical, Westborough, MA, USA) (Table 1).

### 2.2. Cell Viability and ELISA

Human bronchial epithelial cells (BEAS-2B, ATCC, VA, USA) were cultured in DMEM-Ham’s F12 50:50 mixture (Cat#: 1130032 Thermo Fischer Scientific Waltham, MA, USA) supplemented with 5% FBS (Cat#: 10438026, Thermo Fisher Scientific, Waltham, MA, USA), 15 mM HEPES, penicillin (100 U/mL), and streptomycin (100 μg/mL). Cells were cultured to 80% confluency and serum deprived overnight before exposure. For cell viability measurements, cells were seeded in 12 well culture plates and treated with MWCNTs for 24 h, then cells were detached by 0.25% trypsin and neutralized with complete medium. Cells were mixed with equal volume of Viastain^TM^ acridine orange/propidium iodide (AO/PI, Nexelcom Biosciences, Lawrence, MA, USA) and counted on a Cellometer Auto 2000. Absolute cell count and viability were used to determine cytotoxicity. For cell proliferation measurements, cells were grown to 50% confluency in 6 well culture plates, treated for 48 h, and then imaged with an Olympus CKX41 (Center Valley, PA, USA) inverted microscope. Subsequently, the cells were counted as described above. IL-8 was measured in cell supernatants using an Invitrogen IL-8 detection kit (Cat#: CHC1303, Thermo Fisher Scientific, Waltham, MA, USA) and done according to the manufacturers’ instructions. TNF-α (10 ng/mL, Cat#: ab9642, Abcam, Cambridge, MA) was used as a positive control for IL-8 secretion.

### 2.3. Cellular Reactive Oxygen Species (ROS) Production

Cellular ROS production was determined using 2′, 7′dichlorofluorescein diacetate (DCF-DA) fluorogenic probe (Cat#: 287810, EMD Bioscience, CA, USA). Briefly, serum deprived cells were incubated with 5 mM DCF-DA reagent for 45 min at 37 °C. Then the media was aspirated and replaced with MWCNT treatments. Non-DCF-DA treated cells were used for blank subtraction (ΔF) and the data represented as fold change over controls. Fluorescence intensities of cells were measured by Cytation 5 Cell Imaging Multi-Mode Reader (BioTek Instruments, Inc., VT, USA)

### 2.4. Western Blotting

For protein analysis, BEAS-2B cells were plated in 6 well culture dishes and treated with either MWCNT (5 μg/mL) and/or TGF-β for 72 h. Cells were lysed with RIPA buffer containing Halt^TM^ Protease and Phosphatase inhibitor cocktail (Cat#: 78440, Thermo Fischer Scientific, Waltham, MA, USA). Protein concentrations were measured in whole cell lysates by Pierce BCA Protein Assay Kit (Cat#: 23225, Thermo Fischer Scientific, Waltham, MA, USA). Total protein (20 µg) was resolved in a gradient SDS-PAGE gel and transferred to a nitrocellulose membrane. The membrane was incubated with anti-Fn (ab2413, 1:1000, Abcam), anti-PAI-1 (ab182973, 1:1000, Abcam), anti-p21 (ab109199, 1:1000, Abcam), anti-p16 (ab211542, 1:1000, Abcam), anti-γH2A.X (ab2893, 1:1000, Abcam), anti-H2A.X (ab11175, 1:1000, Abcam), anti-E-cadherin (ab11472, 1:1000, Abcam), and anti-vimentin (ab92547, 1:1000, Abcam) primary antibodies overnight at 4 °C. Membranes were incubated with HRP-conjugated secondary anti-rabbit (Cat#1706515, 1:5000, BioRad, Hercules, CA, USA), or anti-mouse (Cat#: 1706516 1:5000, BioRad, Hercules, CA, USA) for chemiluminescence detection using the Bio-Rad ChemiDoc MP imaging system. Image Lab software (v4.1, BioRad, Hercules, CA, USA) was utilized for densitometric quantification of band intensities.

### 2.5. Cellular Senescence Activity Assay

Detection of SA β-gal activity was determined by using a Cellular Senescence Activity Assay (Cat#: ENZ-KIT129, Enzo Life Sciences, Farmingdale, NY, USA). The assay was performed according to the manufacturer’s instructions. Briefly, BEAS-2B cells were seeded into 96 well plates and treated with MWCNTs for 72 h. Following exposure, the cells were washed 1X PBS and were incubated with cell lysis buffer at 4 °C for 5 min. Whole cell lysate was centrifuged and equal volumes of supernatant and 2X assay buffer were combined in a separate 96 well plate for 2 h. Stop solution was added and senescence activity was defined as the fluorescence intensity at 360/465 nm.

### 2.6. Statistical Analysis

Statistical significance was assessed by Tukey’s HSD following one-way ANOVA in GraphPad Prism 7 (La Jolla, CA, USA). Data are represented as means ± standard error of the mean (SEM). A *p* value < 0.05 was considered statistically significant.

## 3. Results

### 3.1. Cellular Responses to MWCNTs in Human Bronchial Epithelial Cells

**Figure 1:**
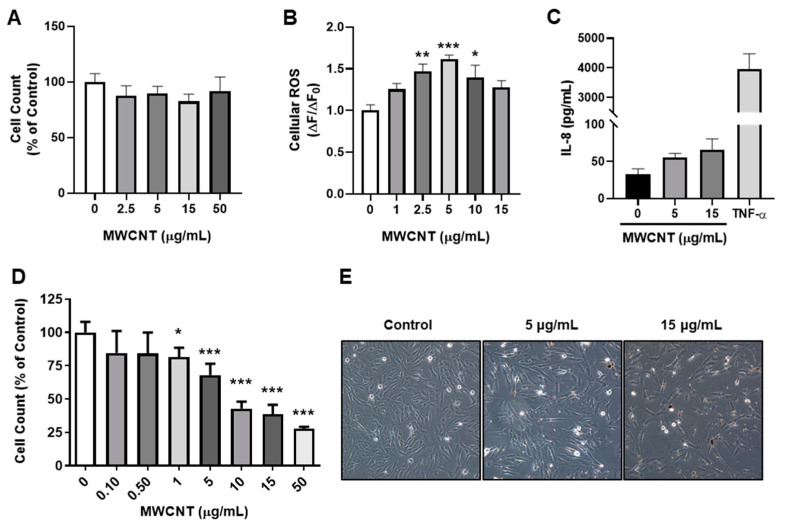
Cellular responses to MWCNT in BEAS2B. (**A**) Cytotoxicity was evaluated by total cell counts 24 h post exposure. Cells were rinsed and stained with AO/PI and then counted in a Cellometer 2000 (*n* = 6). (**B**) Cellular ROS was determined using DCF-DA 8 h post exposure (*n* = 6). (**C**) IL-8 release was assessed by ELISA in MWCNT or TNF-α (10 ng/mL) treated cells 24 h post exposure (*n* = 3). (**D**,**E**) Cell proliferation was assessed by AO/PI staining and light microscopy 48 h post exposure. Data are expressed as mean ± SEM (*n* ≥ 3). Statistical significance was determined by Tukey’s HSD (one-way ANOVA). * *p* < 0.05, ** *p* < 0.01, *** *p* < 0.001 indicates significance. Equal volumes of DPPC in the highest MWCNT concentration tested was added to 0 µg/mL controls.

MWCNTs were evaluated for their cytotoxicity and potential to induce inflammatory and oxidative stress responses. BEAS-2B cells were exposed to MWCNTs for 24 h and then subsequently stained with AO/PI. Cell counts did not change with treatments and displayed no cytotoxicity up to 50 μg/mL, (Figure 1A). In addition, ROS was significantly elevated at 8 h (Figure 1B). Levels of IL-8 trended upwards with increasing MWCNT dose, but were not significantly different (Figure 1C). In cell proliferation assays, MWCNTs led to a dose dependent decrease in cell counts after 48 h at concentrations higher than 0.5 μg/mL (Figure 1D,E).

### 3.2. MWCNT and TGF- β Exposure Propagate Cellular Senescence

**Figure 2:**
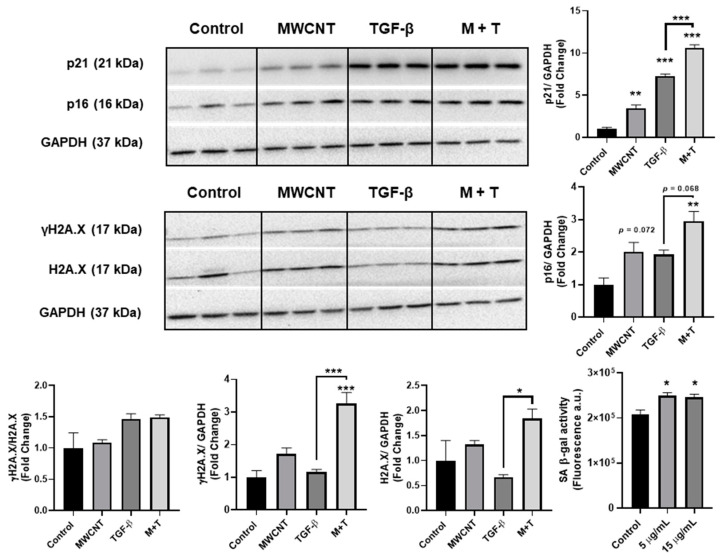
MWCNT and TGF- β exposure propagate cellular senescence. BEAS-2B were exposed to MWCNT (5 μg/mL) and/or TGF-β (5 ng/mL) for 72 h. The activity of SA β-gal and the protein abundance of senescence markers (γH2A.X, H2A.X, p16, and p21) are shown. Data are presented as fold change relative to control. Data are shown as mean ± SEM (*n* = 3/group), * *p* < 0.05, ** *p* < 0.01 *** *p* < 0.01 indicates significance. M + T: Combined MWCNT and TGF-β treatment. An equal volume of DPPC was added to control.

**Figure A1:**
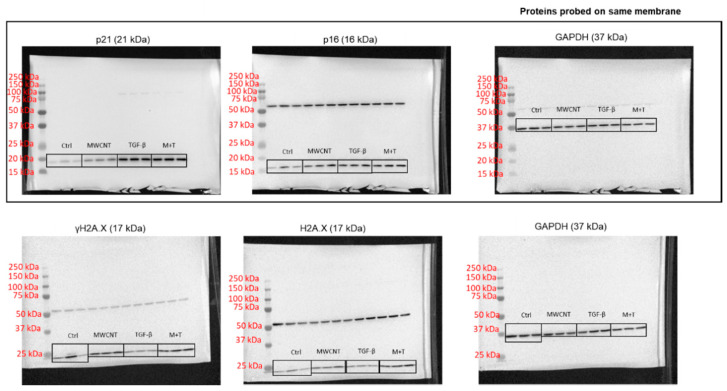
Full unedited gel for Figure 2.

BEAS-2B cells were treated with MWCNTs and/or TGF-β for 72 h, then evaluated for the markers of cellular senescence and cell cycle arrest. MWCNT exposure led to elevated protein levels of SA β-gal, and p21; a non-significant increase in the protein abundance of p16, γH2A.X, and H2A.X was also observed after MWCNT treatment. Co-treatment (MWCNT with TGF-β) resulted in elevated levels of p21, p16, and γH2A.X compared to either TGF-β or MWCNT alone (Figure 2). The full blots are shown in the Appendix A (Figure A1).

### 3.3. MWCNT and TGF- β Induce EMT and Increase ECM Production

**Figure 3:**
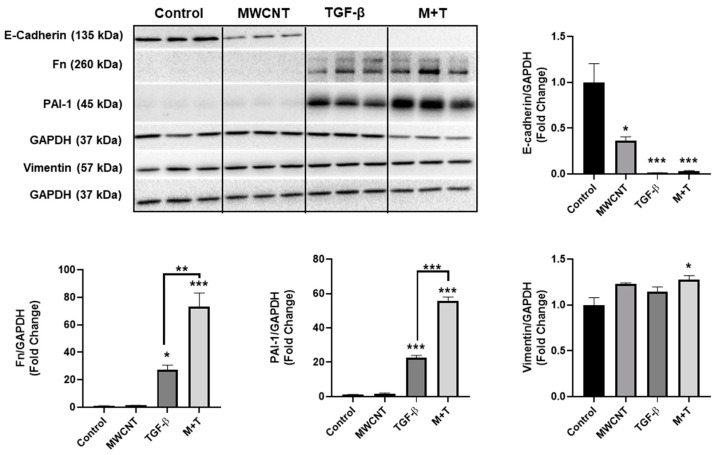
MWCNT and TGF- β induce EMT and increase ECM production. BEAS-2B were exposed to MWCNT (5 μg/mL) and/or TGF-β (5 ng/mL) for 72 h. ECM protein markers were assessed by immunoblot. Data are shown as fold change relative to control; data are shown as mean ± SEM (*n* = 3/group) * *p* < 0.05, ** *p* < 0.01, *** *p* < 0.001 indicates significance. M+T: Combined MWCNT and TGF-β treatment. An equal volume of DPPC was added to control.

**Figure A2:**
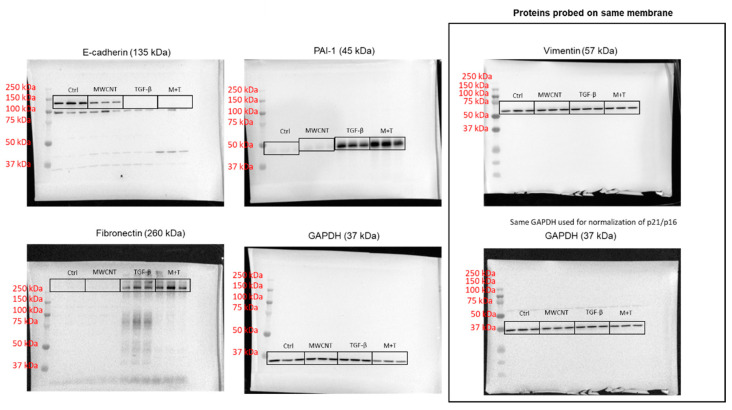
Full unedited gel for Figure 3.

BEAS-2B cells were treated with MWCNTs and/or TGF-β for 72 h and evaluated for the production of EMT and ECM proteins. MWCNT exposure resulted in decreased E-cadherin protein levels (Figure 3). TGF-β alone or the combination of TGF-β + MWCNT dramatically decreased the protein levels of E-cadherin while there is no difference in between the two treatments (Figure 3). For mesenchymal marker measurement, co-treatment of MWCNTs with TGF-β led to an increased protein abundance of PAI-1 and fibronectin compared to TGF-β alone (Figure 3). There is no significant difference in protein levels of vimentin among control, MWCNT, and TGF-β group; while the co-treatment of MWCNT with TGF-β significantly up-regulated the protein abundance of vimentin compared to control (Figure 3). The full blots are shown in the Appendix A (Figure A2).

## 4. Discussion

Multi-walled carbon nanotubes (MWCNTs) were initially studied for their potential fibrogenic activity shortly after their invention in 1991. Due to the propensity of small-scale fibers such as asbestos to cause inflammatory lesions and subsequent fibrotic foci, there was a large concern regarding potential human exposure [23]. Multiple studies have confirmed the fibrogenic potential of certain types of MWCNTs, but only a few studies have examined the effects of MWCNTs on cellular senescence [24,25]. Pulmonary fibrosis is a disease of aging and senescence with a median age of diagnosis of 66 years of age [26,27]. This study sought to evaluate whether exposure to MWCNTs would lead to cellular senescence and pro-fibrotic phenotypes in human bronchial epithelial cells (BEAS-2B). In this study, we demonstrate that MWCNT (5 μg/mL) exposure leads to increased cellular ROS production, a reduction of cellular proliferation, and elevated levels of senescence markers (p21 and SA β-gal). Moreover, co-exposures with a fibrotic stimulus, TGF-β (5 ng/mL), produced a more severe senescence phenotype, suggesting that MWCNTs induced senescence may be dependent on the cellular microenvironment.

Senescent cells accumulate naturally during aging in response to a number of external and internal stimuli such as DNA damage, metabolic alterations, and elevated ROS production [28]. Previous studies have reported that MWCNTs induce oxidative stress and can exhaust the antioxidant cellular capacity [29,30]. Our results are consistent with these findings and demonstrate that MWCNTs are capable of disrupting redox biology, resulting in increased production of ROS in a dose dependent manner. At higher doses, we observed a decline in ROS production. While a non-monotonic dose–response is possible, the decreased response is likely due to increased interference of the fluorescence signal by MWCNTs. This would suggest that ROS production at higher MWCNT doses is underestimated and should be taken into consideration when interpreting our results. Exposure of sub-confluence cultures of BEAS-2B cells to MWCNTs over 48 h resulted in cell growth arrest and inhibition of cellular proliferation. Evidence indicates that elevated levels of ROS may be required for certain types of premature senescence. In a previous report, induction of the cyclin dependent kinase (CDK) inhibitor p21 produced a robust increase in ROS, resulting in cellular senescence in EJ cells. Moreover, N-acetyl-L-cysteine, a potent antioxidant can prevent p21 induced ROS accumulation and subsequent senescence [31,32]. IL-8 was not significantly changed but trended upwards. It is possible that other cytokines may be involved in the induction of cellular senescence. In addition, crosstalk with resident and recruited immune cells may propagate inflammatory responses. Here, we show that exposure to MWCNTs leads to increased oxidative stress, an important factor mediating cellular senescence.

To further evaluate whether MWCNTs were leading to the development of cellular senescence we correlated our findings with established senescence biomarkers. Our data indicate that MWCNT exposure recapitulates some of the features of classical senescence such as an increase in SA β-gal activity and an increase in the protein abundance of p21 and a slight increase in p16, but there was no change in γH2A.X, a sensitive indicator of DNA double-strand (ds) breaks. In response to DNA ds breaks, Ataxia Telangiectasia Mutated (ATM) and other kinases phosphorylate and recruit H2A.X to ds breaks. This subsequently recruits other DNA repair mechanisms and triggers downstream p53/p21 CDKs to halt cell cycle progression. [33]. Accumulation of γH2A.X has been correlated with telomere shortening and induction of senescence. However, γH2A.X is observed in response to all ds breaks and does not necessarily converge to irreversible cell cycle arrest. Interestingly, many MWCNTs display little to no mutagenicity, possibly due to the induction of compensatory DNA repair mechanisms [24]. However, TGF-β can suppress DNA double strand repair mechanisms which is hypothesized to aid in genetic diversity in immune cells [34]. As a consequence, TGF-β may facilitate the mutagenic potential of MWCNTs. Indeed, co-exposure with MWCNTs resulted in an exacerbated senescence response with a significant increase in p16, p21, and γH2A.X protein levels compared to TGF-β alone. The effects of TGF-β and MWCNT on p21 and p16 appear to be additive, but the combined effects on γH2A.X suggest that synergistic effects may occur. TGF-β is known to induce cellular senescence and is sufficient to induce pulmonary fibrosis in vivo [35,36]. MWCNT can activate SMAD proteins downstream of TGF-β [37] and may work in concert with added TGF-β to overstimulate the induction of senescence, but more research is needed to clarify these results. Whether the observed combinatory effect of MWCNTs and TGF-β is a consequence of a unique interaction between them or results from the breakdown of normal repair machinery due to multiple hits requires more attention.

To further investigate the potential combinatorial effects of TGF-β and MWCNTs, the EMT was evaluated. During EMT, epithelial cells lose cell polarity and contact adhesion molecules such as E-cadherin. Although, there is not a clear consensus on the exact role of EMT in pulmonary fibrosis, evidence suggests that EMT may either supply the proliferation of myofibroblasts and/or produce mesenchymal support cells that stimulate the production of ECM proteins by activated myofibroblasts [38]. Previous reports indicate that MWCNTs can lead to a significant loss of E-cadherin [13], consistent with our findings. However, MWCNTs failed to increase the production of ECM proteins fibronectin or PAI-1. Conversely, co-exposure of MWCNTs with TGF-β led to a marked increase in both fibronectin and PAI-1 compared to TGF-β alone. Interestingly, PAI-1 protein levels appear to be sensitive to the combination treatment. PAI-1 is a serine protease downstream of p53 and has been identified as a key mediator of cellular senescence. Fibroblasts deficient in PAI-1 are resistant to cellular senescence [39] and knockdown of PAI-1 blunts TGF-β induced senescence [40]. Production of PAI-1 and fibronectin by MWCNT and TGF-β treated cells may be indicative of a breakdown of homeostatic repair mechanisms and senescence checkpoints. Collectively, these data suggest that MWCNT induced senescence may in part be dependent on the cytokine environment of the lung and that pro-fibrotic stimuli may interact with MWCNTs to propagate adverse cellular effects.

In conclusion, our study highlights the potential of MWCNTs to induce cellular senescence and provides new insight into its possible interactions with other senescent stimuli. While MWCNTs alone were only able to recapitulate some of the features of senescence, co-exposure with TGF-β resulted in a dramatic increase in cellular senescence and ECM production. Based on our data, the dysregulation of TGF-β signaling may promote MWCNT induced cellular senescence, which may be an underlying event in MWCNT-induced fibrotic responses.

## Figures and Tables

**Table 1:** MWCNT Physical Characteristics.

**MWCNT Characteristics**	
Length *	10–30 µM
Outer Diameter *	<8 nm
Z-average	146.1 nm
PdI	0.419
Zeta Potential	−10.7 mV

* Measurements were provided by the manufacturer.

## Data Availability

We declare that we have provided all the data, but the primary data will be available upon request. Full unedited and uncropped blots are provided in the Appendix A.
